# Examining Epigenetic Age in Women with Different Obesity Conditions Using DNA Methylation at the *FHL2* Gene

**DOI:** 10.3390/mps9020047

**Published:** 2026-03-12

**Authors:** Licínio Manco, Helena Correia Dias, Lara Palmeira

**Affiliations:** 1 Research Centre for Anthropology and Health (CIAS), Department of Life Sciences, University of Coimbra, 3000-456 Coimbra, Portugal; 2 National Institute of Legal Medicine and Forensic Sciences, 3000-548 Coimbra, Portugal; 3 RISE-Health, Department of Psychology and Education, Portucalense University, 4200-072 Porto, Portugal; 4 Faculty of Psychology and Educational Sciences, Center for Research in Neuropsychology and Cognitive and Behavioural Intervention (CINEICC), University of Coimbra, 3000-115 Coimbra, Portugal

**Keywords:** DNA methylation, age prediction, epigenetic age acceleration, obesity, BMI, droplet digital PCR

## Abstract

DNA methylation (DNAm) age estimation is one of the hottest topics in forensic contexts. However, there is growing evidence that DNAm can be affected by several factors, including many clinical conditions. In this study, we analyzed the methylation levels within the *FHL2* gene in Portuguese women using the droplet digital PCR (ddPCR) methodology to develop age prediction models (APMs). We hypothesized that obesity could affect the accuracy of APMs and would be associated with the advancement in epigenetic aging. We collected blood samples from 62 women (aged 21–58 years old) with overweight and obesity. DNA extracts were subjected to bisulfite conversion followed by ddPCR using dual-labeled probes targeting the methylated and unmethylated *FHL2* CpG site cg06639320. The developed APM yielded a mean absolute deviation (MAD) of 4.72 years between predicted and chronological ages in the total sample. When applying the developed APM to women classified as overweight, the MAD was 3.64 years, while, for those with obesity class 1, it was 3.93 years, and, for those with obesity class 2, 6.29 years. The same pattern of accuracy was observed when we developed APMs specifically for the groups categorized by overweight and obesity, obtaining MAD values of 3.75 years (overweight), 3.69 years (obesity class 1) and 6.24 years (obesity class 2). Our study indicates that severe obesity may impact the accuracy of DNA methylation-based age estimators. We did not find evidence of an association between BMI and accelerated epigenetic aging. However, we found signals of epigenetic age acceleration in younger subjects and epigenetic age deceleration in the older participants.

## 1. Introduction

Obesity is defined as abnormal or excessive fat accumulation and is a risk factor for the development of several chronic diseases [1]. This condition affects individuals of all age groups, and it is considered a consequence of unhealthy behavioral exposures, such as physical inactivity and excessive energy intake, in association with a strong genetic background [2,3].

In the last decade, DNA methylation (DNAm) has been proposed as a possible contributor to the etiology of obesity [4]. Several epigenome-wide studies evidenced the association between DNAm and obesity-related traits such as body mass index (BMI) [5,6]. Other studies of obesity-related genes (like *SIM1*, *POMC*, *MC4R*) showed a link between DNAm and BMI, suggesting that methylation patterns in genes controlling metabolism and appetite reflect and influence obesity risk [7]. However, some findings suggest that BMI drives methylation changes more than the reverse, highlighting the complex interplay of genes, environment, and early life factors in obesity [5].

DNAm is the enzymatic incorporation of a methyl group on the 5′ carbon of cytosines followed by guanine (CpG), located mainly at gene promoters but also in other regions of the genome [8]. This process is regulated by DNA methyltransferases that add and remove the methyl groups. The advent of DNA methylation array technology enabled the identification of millions of CpG dinucleotides in the human genome shown to have a correlation with chronological age [9]. Following this, several epigenetic age estimators, often referred to as “epigenetic clocks”, have been proposed in a combination of a few to several hundred of these CpG sites to be used for predicting chronological age [10,11].

Many pathophysiological biomarkers, environmental factors, age-related conditions and diseases have been reported to affect DNAm-based age estimation [12]. These deviations between DNAm age and chronological age lead to a concept known as epigenetic age acceleration, which refers to the situation where an individual’s epigenetic age exceeds their chronological age, given a specific metabolic condition, reflecting the impact of such a condition on epigenetic age [13]. This phenomenon was also investigated in obesity using “epigenetic clocks” based on DNAm arrays, revealing a link between BMI and greater epigenetic age across multiple body systems, including metabolically active tissues such as the liver [14] and adipocytes [15], but also saliva [16] and blood [15,16,17,18,19]. However, the findings in blood are not consistent across all studies [17,18,19].

Minimized “epigenetic clocks”, which rely on fewer CpGs, were developed in the last decade for forensic contexts, particularly using the blood samples of healthy individuals. However, most of these APMs have yet to be tested and validated in clinical conditions. Research on DNAm age estimation has primarily focused on six key genes: *ELOVL2*, *KLF14*, *TRIM59*, *FHL2*, *PDE4C*, and *MIR29B2CHG*. This has led to the creation of effective APMs, establishing epigenetic age with a strong correlation with chronological age. Among these genes, *FHL2* (four and a half LIM domains 2) is notably hypermethylated with age, with several critical CpG sites used for accurate age estimation, in particular the CpG site cg06639320 [20]. The FHL2 protein is a key regulator of intracellular signal transduction pathways and subsequent gene regulation. It is able to regulate gene expression via interactions with transcription factors and their upstream co-regulators [20].

Recently, we employed droplet digital PCR (ddPCR) to assess the DNAm profile of the three age-associated genes—*ELOVL2*, *FHL2*, and *PDE4C*—in blood-derived DNA samples from 58 healthy Portuguese individuals to develop simple and multiple regression APMs [21]. The strongest correlation with age was identified for the CpG site cg06639320, located within the *FHL2* gene, with a correlation coefficient of r = 0.948. The age prediction based on the coefficients from the simple linear regression demonstrated a mean absolute deviation (MAD) of 6.028 years between the predicted and the chronological ages. In this study, we aimed to investigate the impact of obesity on the accuracy of age-predictive models by evaluating methylation levels at the *FHL2* gene CpG site cg06639320 in blood samples. Additionally, we explored the relationship between BMI and the progression of epigenetic aging.

## 2. Materials and Methods

### 2.1. Study Population

This study included a total sample of 62 unrelated Portuguese women, aged 21–58 years old (mean 42.26), with the following body mass index (BMI) classifications: 18 individuals with overweight (25 kg/m^2^ ≤ BMI < 30 kg/m^2^; mean 27.77); 21 individuals with obesity class 1 (30 kg/m^2^ ≤ BMI < 35 kg/m^2^; mean 32.24); 23 individuals had second-degree obesity class 2 (BMI ≥ 35 kg/m^2^; mean 38.82). This study population was a subsample of that previously analyzed by Palmeira et al. [22]. For comparison purposes, we used DNAm data from blood samples of 25 healthy women of the general Portuguese population, aged 21–58 years, although with unknown BMI. This subset is part of a larger sample analyzed in a prior study [21].

### 2.2. DNA Extraction and Bisulfite Conversion

Peripheral blood was collected from each individual, and genomic DNA extraction was performed using a QIAamp DNA Mini Kit (Qiagen, Hilden, Germany). DNA extracts were quantified with a NanoDrop spectrophotometer (Thermo Fisher Scientific, Waltham, MA, USA). The subsequent bisulfite conversion of 20 μL of genomic DNA (in a total amount of 200 to 400 ng) was performed using an EZ DNA Methylation Gold Kit (Zymo Research, Irvine, CA, USA), according to the instructions of the manufacturer, which produced a final volume of 10 μL of modified DNA.

### 2.3. Droplet Digital PCR (ddPCR)

After bisulfite conversion, the modified DNA was submitted to a ddPCR assay in a QX100 Droplet Digital PCR System (Bio-Rad, Hercules, CA, USA) as previously described in [22]. Each reaction mixture contained 10 μL of ddPCR Supermix for probes (no dUTP) (Bio-Rad), 0.5 μM each of the forward and reverse primers, 0.25 μM of TaqMan dual-labeled probes targeting the methylated and the non-methylated sequences, and 1 μL of bisulfite-converted DNA, for a total amount of 20 μL. Subsequently, the reaction was emulsified with 70 μL of Droplet Generator Oil (Bio-Rad) in a DG8 Cartridge (Bio-Rad) using a QX100 Droplet Generator (Bio-Rad). The droplets (40 μL) were then transferred to a 96-well reaction ddPCR plate (Bio-Rad), and the plate was heat-sealed with PX1 PCR Plate Sealer (Bio-Rad) for amplification using a CFX96 Touch Thermal Cycler (Bio-Rad). The PCR protocol was: 95 °C 10 min followed by 40 cycles of 95 °C 30 s and 56 °C 1.30 min (2.0 °C/s ramp rate) with a 10 min step at 98 °C for enzyme deactivation and a final hold at 4 °C. The primer sequences and dual-labeled probes targeting the methylated and unmethylated *FHL2* CpG site cg06639320 are described in Han et al. [23]. After PCR amplification, droplets were read using a QX100 Droplet Reader (Bio-Rad), and fluorescence data were analyzed using Quanta-Soft software (version 1.7.4) (Bio-Rad). Samples with more than 10,000 droplets (including at least 100 positive droplets for both FAM and HEX probes) were considered for further analysis.

### 2.4. Statistical Analysis

The methylation levels at the *FHL2* CpG site for each sample were determined by calculating the ratio of the measured concentration (copies/μL) of methylated to unmethylated droplets through the formula M/(M + U), where M represents the percentage of methylated CpG, and U represents the percentage of unmethylated CpG. The predicted age was calculated using the obtained simple linear regression coefficients from the DNAm levels for chronological age according to the formula: predicted age (years) = a + bxi, where a is the intercept, b is the slope, and xi is the obtained methylation value for each sample. The normality of the dependent variable, chronological age, was assessed by the Kolmogorov–Smirnov test. The accuracy of the developed model was evaluated by the mean absolute deviation (MAD) between predicted and chronological ages. To assess the model performance in the total sample of 62 women, a 4-fold cross-validation was used, where a set of samples (about 25%) was randomly removed four times from the total training set to develop four independent linear regressions on the remaining samples. The set of removed samples was used for validation purposes, estimating the MAD values for each of the four independent linear regressions. For the groups established by BMI categories and for the control sample, the model performance was assessed by holdout cross-validation, in which the complete data set was split into two sets (training and validation sets). An independent regression was conducted on the training set, and the developed predictive model was subsequently applied to the validation set. Age acceleration for BMI was investigated by estimating correlation coefficients (Pearson’s r) between the BMI variable and the deviation values (residuals) between predicted and chronological ages (predicted age minus chronological age). To determine differences in continuous variables between groups, we used the Kruskal–Wallis test. Data and graphical analyses were conducted using the statistical package IBM SPSS Statistics for Windows, version 27 (IBM Corp., Armonk, NY, USA).

## 3. Results

In this study, we employed ddPCR to analyze the methylation profile of the CpG site cg06639320 within the age-associated gene *FHL2* in blood-derived DNA samples from 64 Portuguese women aged between 21 and 56 years with overweight or obesity. The obtained methylation values at the *FHL2* CpG site are detailed in the Supplementary Table S1. Sample characteristics are shown in Table 1.

### 3.1. Development of an Age Prediction Model for the Total Sample

**Figure 1:**
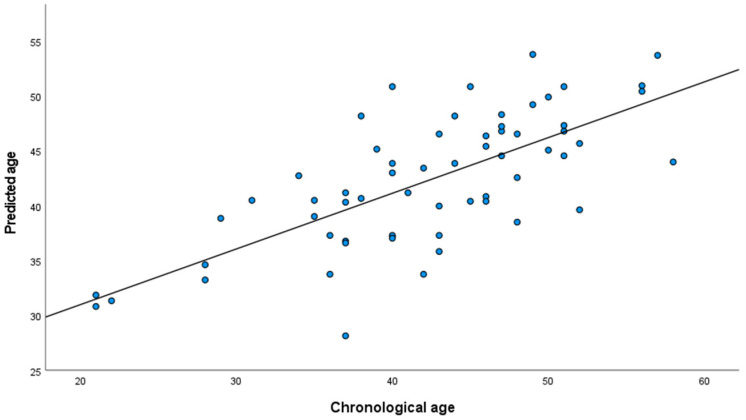
Scatterplot showing the relationship between the epigenetic and chronological ages.

**Figure 2:**
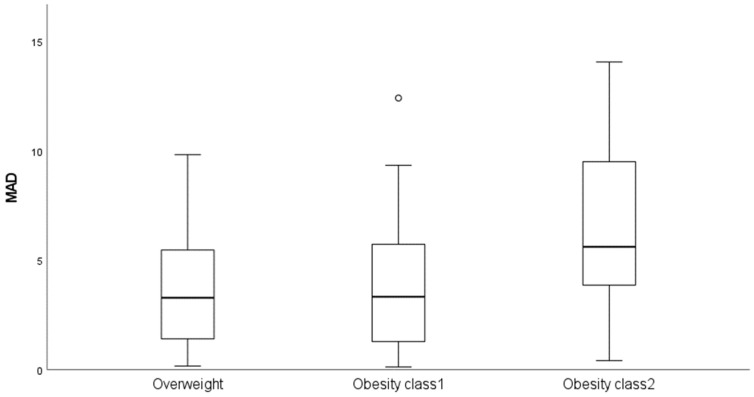
Boxplots showing the distribution of the mean absolute deviations (MAD) values between the epigenetic and chronological ages in the three phenotypic groups.

The methylation levels at the *FHL2* CpG site cg06639320 and chronological age demonstrated a positive linear correlation (Figure S1). In the total sample, the simple linear regression from the DNAm levels on chronological age revealed a strong correlation (r = 0.712; *p* = 8.596 × 10^−13^), accounting for approximately 51% of the variance in age (Table 2). The predicted age for each individual (detailed in Table S1) was obtained using simple linear regression coefficients. The obtained MAD value between the predicted and chronological ages was 4.72 years, with a Pearson’s correlation between predicted and chronological ages of 0.712 (*p* = 8.596 × 10^−11^) (Figure 1). The accuracy of the model was tested by 4-fold cross validation. The mean MAD value obtained amongst the four validation sets was 4.96 years, which was very close to the MAD of 4.72 years from the overall population. We applied the predictive model to the sample based on phenotype, obtaining MAD values of 3.64 years for women with overweight, 3.93 years for women with obesity class 1, and 6.29 years for those with obesity class 2 (see Table 2 and Figure 2), reaching a significant difference between groups in the Kruskal–Wallis test (*p* = 0.028).

### 3.2. Development of Age Prediction Models by BMI Categories

We developed separate APMs specifically for the groups categorized as overweight and obesity class 1 and class 2. In the overweight group, we found a strong correlation (r = 0.79; *p* = 0.000095) between DNAm levels and chronological age, explaining approximately 62% of the variance in age (Table 2). The APM derived from the simple linear regression coefficients indicated a MAD from chronological age of 3.75 years. The obesity class 1 group exhibited a strong correlation between DNAm and age (r = 0.764; *p* = 0.000056), accounting for about 58% of the variance in age (Table 2). The APM for this group allowed us to estimate a MAD from the chronological age of 3.69 years. In contrast, the obesity class 2 group exhibited a moderate correlation between DNAm and age (r = 0.597; *p* = 0.003), accounting for about 36% of the variance in age (Table 2). The APM for this group allowed us to estimate a MAD from the chronological age of 6.24 years. The accuracy of the models was tested by using holdout cross-validation, splitting the total sample set of each group into two subsets (training and validation). For the groups categorized as overweight, obesity class 1, and obesity class 2, MAD values of 4.43 years, 3.82 years, and 5.81 years were obtained for the training sets, and MAD values of 3.89 years, 5.16 years, and 6.73 years were obtained for the validation sets, respectively.

### 3.3. Analysis of DNAm Age Acceleration

**Figure 3:**
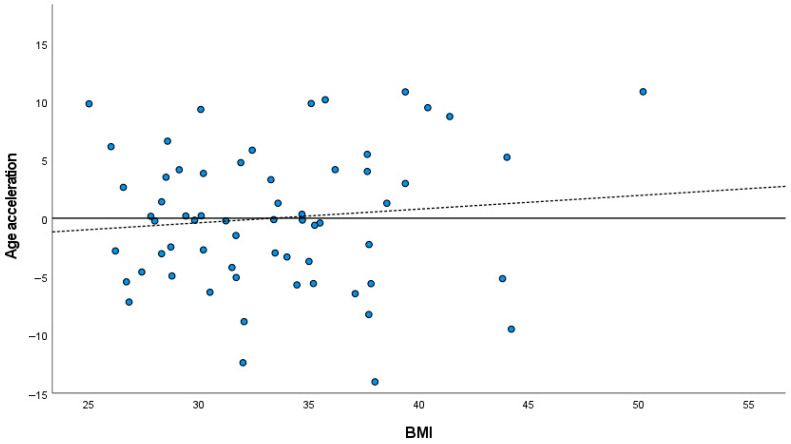
Scatterplot showing the relationship between the deviation values (residuals) between the predicted and chronological ages and body mass index (BMI). The dashed line is the line of regression that models the relationship between residuals and BMI; the horizontal line represents a reference regression coefficient (slope) of zero.

We defined age acceleration based on the deviation values (residuals) between predicted and chronological ages. No significant age acceleration for BMI was observed for the total sample (Pearson’s correlation coefficient r = 0.104; *p* = 0.423) (Figure 3) as well as for women with overweight (r = −0.168; *p* = 0.505), obesity class 1 (r = −0.109; *p* = 0.638) and class 2 (r = 0.174; *p* = 0.426). Of note, the estimated epigenetic age exceeded the chronological age (MAD = 7.93 years) in all subjects aged ≤35 years (*n* = 10; 16.1%); conversely, in all subjects aged ≥50 years (*n* = 12; 19.4%), the epigenetic age was lower than the chronological age (MAD = 5.54 years).

### 3.4. Development of an Age Prediction Model in a Reference Sample of Women

We developed an APM based on the blood of 25 healthy age-matched women from the general population, with unknown BMI, focusing on the DNAm data of the same *FHL2* CpG site cg06639320. The obtained age correlation value (r) was 0.629 (*p* = 0.001), and the regression coefficients allowed us to predict age with a MAD from the chronological age of 5.84 years. Using holdout cross-validation, by splitting the whole sample set into two subsets (training and validation), a MAD of 6.29 years was obtained for the training set and a MAD of 5.81 years for the validation set.

## 4. Discussion

In the current study, we analyzed the CpG site cg06639320 within the FHL2 gene in the blood from 62 Portuguese women aged 21–56 years old, categorized as overweight and obesity class 1 and class 2. We used ddPCR to evaluate the DNAm levels for developing APMs. We hypothesized that obesity would affect the accuracy of the age prediction model and would be associated with the advancement of epigenetic aging.

The regression coefficients allowed for predicting age with a MAD from the chronological age of 4.72 years for the entire sample. A similar value was obtained for an independent control sample of age-matched healthy women from the general population (MAD = 5.84 years), suggesting that BMI does not alter the accuracy rate of the epigenetic clock. However, predicted ages with different MAD values were estimated for women with overweight (3.64 years), obesity class 1 (3.93 years), and obesity class 2 (6.29 years), reaching a significant difference between the three groups (*p* = 0.028). A similar trend in accuracy was observed when developing the APM specifically for groups classified by BMI categories: for women with overweight, the MAD from chronological age was 3.75 years; in women with obesity class 1, the MAD was 3.69 years; and, for those with obesity class 2, the MAD increased to 6.24 years. Therefore, we observed lower accuracy in the APM for women with obesity class 2 compared to those classified as overweight or having obesity class 1 or from the general population. Our findings suggest that the prediction accuracy varies based on the individual’s BMI, indicating that BMI influences the methylation process. In fact, the reduced accuracy of the APM in women with obesity class 2 aligns with the lower correlation observed between chronological age and DNAm (r = 0.597). In contrast, women classified as overweight and obesity class 1 had higher correlation values (r = 0.79 and r = 0.764, respectively).

The currently developed forensic epigenetic age estimators, often referred to as “epigenetic clocks”, using a combination of a few to several hundred of CpG sites, are mostly trained on blood of “healthy or normal-weight” individuals. Our present results, suggesting that the severe obesity condition affects the accuracy of APMs, raise concerns about “bias” if such clocks are used in clinical or forensic settings or to compare individuals of differing body compositions without adjusting for obesity-related variables. The higher MAD value in the second-degree obesity group observed in our results suggests that the APMs established based on DNAm under- or over-estimates biological age for individuals with obesity, particularly if the tissue sampled or methylation signature is sensitive to BMI or related metabolic factors.

Epigenetic age acceleration, i.e., the deviation of epigenetic age from chronological age, given a specific metabolic condition, reflects the impact of such condition on epigenetic age. Recent evidence strengthens the notion that obesity (especially long-term or severe) accelerates epigenetic aging as measured by DNAm clocks. This effect was observed in metabolically active tissues, such as the visceral adipose tissue [15] and the liver [14], but also in saliva cells [16]. Interestingly, this positive correlation between BMI and epigenetic age acceleration was not validated by these studies in blood or in other tissues, such as muscle or subcutaneous adipose tissue [14,15].

Our results, showing no significant age acceleration for BMI, are consistent with those studies where a positive correlation between BMI and epigenetic age acceleration was not observed in the blood [14,15]. However, other factors besides the tissue type can confound the relationship between BMI and age acceleration, including factors such as the selected clock, severity of obesity, age, or sex, which can explain why the results obtained in blood are not consistent among studies. A long-term obesity study in young adults using DNA from blood showed that individuals with obesity since adolescence (or early childhood) had a higher DNAm age (by the Horvath clock and GrimAge clock) compared to those who remained at normal weight—sometimes with biological age up to ~48% higher than chronological age [19]. Also, a study in middle-aged participants using DNA from blood showed a significant positive correlation between BMI and an increased epigenetic age using the Horvath algorithm based on the methylation level of 353 CpG sites [17]. In another study based on DNAm data from blood, long-term obesity (but not overweight) was associated with epigenetic age acceleration in older adults, particularly among individuals with low or moderate genetic risk for obesity [18].

Interestingly, in our study, we found that the estimated epigenetic age exceeded the chronological age in younger subjects aged ≤35 years, suggesting an epigenetic age acceleration. Conversely, in the older individuals in the study sample (≥50 years), the epigenetic age was lower than the chronological age, which suggested slower biological aging, a phenomenon referred to as epigenetic age deceleration. These results align with those obtained in children, where biological age was higher than actual age, and this difference was explained by early-life adversity, psychosocial stressors, environmental exposures, and the rapid development of the immune system [24,25]. In contrast, individuals of advanced age (e.g., centenarians and supercentenarians) consistently exhibit a younger epigenetic age compared to their chronological age [26].

In this study, we focused on a single biomarker to assess epigenetic aging. The *FHL2* CpG site (cg06639320) that we examined has been identified as one of the most hyper-methylated markers associated with aging, demonstrating a consistent linear trend across various tissues, including blood, saliva, and buccal swabs [20]. In a previous study [21] examining the blood samples of healthy individuals from the general population using three DNAm markers with the same methylation detection technique, we found that the *FHL2* cg06639320 CpG site exhibited a strong correlation with chronological age (r = 0.948), comparable to the widely recognized epigenetic biomarker ELOVL2 (r = 0.926), and higher than PDE4C (r = 0.819). The single regression coefficients allowed us to predict age with a MAD of 6.028 years from the chronological age for the *FHL2* CpG site. This accuracy was greater than that of the single models for PDE4C (MAD = 8.667 years) and ELOVL2 (MAD = 7.295 years), although slightly lower than the multivariable model that utilized DNAm levels for three CpG sites (MAD = 4.657 years). We hypothesize that the results obtained in the present study from the single *FHL2* CpG site (cg06639320) effectively represent the impact of obesity on DNA methylation-based age biomarkers. However, we acknowledge that using a multivariable age prediction model that incorporates multiple CpG sites could enhance predictive accuracy and provide a more comprehensive understanding of this influence.

The present study has some limitations, including the fact that the study population comprised women only. This factor may have affected the relationship between obesity and accelerated methylation age, which appeared to depend on various factors, including the characteristics of the study population (such as age, ancestry, and sex). The use of a control group consisting of women with no known BMI is also a major drawback since it was not possible to ensure a comparison of the accuracy of APMs with a normal-weight phenotypic class. Another significant limitation of this study is the small sample size, particularly for the groups categorized as overweight, obesity class 1, and obesity class 2. However, we must note that the normality of the dependent variable, chronological age, was confirmed using the Kolmogorov–Smirnov test, and the sample size per group was not far from the robust minimum for regression (*N* ≥ 25). Moreover, the validation performance of each developed regression model allowed us to obtain MAD values from the validation sets that were generally very close to the MAD values obtained from the entire training sets, thus revealing the accuracy and reproducibility of the APMs in each cluster. Despite this, larger sample sets could have higher statistical power and be more representative of DNAm across different age ranges or BMI categories. The reliance on a single DNAm marker is also a significant limitation. This was mainly due to logistical issues that limited the testing of additional markers. We recognize that including more methylation biomarkers could improve predictive accuracy in a multivariable regression model, allowing for a deeper understanding of how obesity affects DNAm-based age estimation. DNAm can also be influenced by participants’ lifestyle factors, such as the medications they use, tobacco consumption, physical activity, and diet, among others. Unfortunately, we did not have access to this information in this study.

## 5. Conclusions

Because people with obesity are at an increased risk of many age-related diseases, it is a plausible hypothesis that obesity increases the biological age of some tissues and cell types. In our study, we investigated epigenetic age as predicted by the *FHL2* CpG site cg06639320 in women with overweight and obesity using ddPCR technology to quantify methylation. The results suggest that severe obesity affects the accuracy of DNAm-based age estimators. We did not find evidence of an association between BMI and accelerated epigenetic aging. However, we found signals of epigenetic age acceleration in younger subjects and epigenetic age deceleration in the older participants. Future research that utilizes additional CpG markers from other age-related genes in larger sample sizes has the potential to strengthen these findings.

## Figures and Tables

**Table 1:** Characteristics of the study population for the total sample and by phenotype condition.

Parameters	Total	Overweight	Obesity Class 1	Obesity Class 2	*p*
*N*	62	18	21	23	
Age Mean (range)	42.26 (21–58)	41.61 (21–51)	45.24 (22–57)	40.04 (21–58)	
BMI mean (SD)	33.39 (5.22)	27.77 (1.31)	32.24 (1.56)	38.82 (3.76)	**<0.0001**
BMI median (IQR)	32.85 (8.22)	28.15 (2.07)	32 (2.67)	37.73 (4.66)	
Mean Chronological Age	42.26 (8.41)	41.61 (7.52)	45.24 (7.74)	40.04 (9.16)	0.052
Median Chronological Age	43.0 (11.0)	43.0 (9.0)	47 (11)	38 (11)	
Mean Epigenetic Age	42.26 (5.99)	41.81 (5.39)	44.06 (6.93)	40.97 (5.32)	0.171
Median Epigenetic Age	42.63 (9.49)	41.85 (8.39)	46.34 (10.64)	40.47 (7.25)	
Mean MAD Score (SD)	4.72 (3.49)	3.64 (2.74)	3.93 (3.36)	6.29 (3.68)	**0.028**
Median MAD Score (IQR)	4.2 (4.44)	3.28 (4.51)	3.33 (4.96)	5.61 (5.8)	
Mean Methylation Levels (SD)	43.07 (6.94)	42.56 (6.24)	45.16 (8.03)	41.58 (6.16)	0.171
Median Methylation Levels (IQR)	43.5 (11.0)	42.6 (9.72)	47.8 (12.32)	41 (8.4)	

Abbreviations: *N*, number of individuals; BMI, body mass index; MAD, mean absolute deviation between chronological and predicted ages. Data are presented as *n* (%), mean ± SD, or median (interquartile range, IQR). The Kruskal–Wallis test was used for comparison of quantitative variables between groups (overweight, obesity class 1 and obesity class 2). Significant *p*-values are in bold.

**Table 2:** Simple linear regression statistics testing the association between the methylation values of the CpG cg06639320 at the *FHL2* gene and chronological age.

	*N*	Slope	Intercept	r	*p* Value	r^2^	MAD	MAD OW	MAD Ob Class 1	MAD Ob Class 2
Total Sample	62	0.863	5.088	0.712	8.596 × 10^−13^	0.507	4.72	3.64	3.93	6.29
Overweight	18	0.952	1.116	0.79	0.000095	0.624	3.75			
Obesity Class 1	21	0.736	12.017	0.764	0.000056	0.583	3.69			
Obesity Class 2	23	0.887	3.164	0.597	0.003	0.356	6.24			
Reference Sample	25	1.026	−0.462	0.629	0.001	0.396	5.84			

Abbreviations: *N*, number of individuals; r, Pearson’s correlation coefficient; SE, standard error; MAD, mean absolute deviation between chronological and predicted ages; OW, overweight; Ob, obesity.

## Data Availability

The original contributions presented in this study are included in the article/Supplementary Materials. Further inquiries can be directed to the corresponding author.

## Supplementary Materials

The following supporting information can be downloaded at: https://www.mdpi.com/article/10.3390/mps9020047/s1, Figure S1: Scatterplots of DNAm levels of the *FHL2* CpG site cg06639320 with chronological age for women with overweight and obesity; Table S1: DNA methylation profiles at the *FHL2* CpG site cg06639320 obtained by ddPCR of 62 blood samples from women with overweight and obesity, and deviation values (residuals) between predicted and chronological ages.
