# Supplementary material for: Examining Epigenetic Age in Women with Different Obesity Conditions Using DNA Methylation at the FHL2 Gene
Source: Methods Protoc. 2026 Mar 12;9(2):47. doi: 10.3390/mps9020047 (PMC13010738; doi:10.3390/mps9020047)
Supplement: Supplementary file 1 [file mps-09-00047-s001.zip › mps-4152844-supplementary.pdf]

SUPPLEMENTARY MATERIALS

Table S1. DNA methylation profiles at the *FHL2* CpG site cg06639320 obtained by droplet digital PCR (ddPCR) of the 62 blood samples from women with overweight and obesity, and deviation values (residuals) between predicted and chronological ages.

ID	Chronological age	BMI	FHL2 Methylation	Predicted age	Age deviation
1	31	40.4	41	40.471	9.471
2	37	35.5	36.5	36.5875	-0.4125
3	52	30.5	47	45.649	-6.351
4	51	29.8	53	50.827	-0.173
5	37	36.19	41.8	41.1614	4.1614
6	51	31.5	48.3	46.7709	-4.2291
7	40	30.2	44.9	43.8367	3.8367
8	48	44.2	38.7	38.4861	-9.5139
9	44	34.7	44.9	43.8367	-0.1633
10	50	33.4	51.9	49.8777	-0.1223
11	52	32	40	39.608	-12.392
12	42	28.3	44.4	43.4052	1.4052
13	29	35.1	39.1	38.8313	9.8313
14	56	31.69	53.1	50.9133	-5.0867
15	44	29.1	49.9	48.1517	4.1517
16	43	28.3	40.4	39.9532	-3.0468
17	40	26.2	37.2	37.1916	-2.8084
18	40	30.2	37.3	37.2779	-2.7221
19	39	26	46.4	45.1312	6.1312
20	51	35	48.9	47.2887	-3.7113
21	45	27.4	40.9	40.3847	-4.6153
22	51	37.1	45.7	44.5271	-6.4729
23	48	26.7	43.4	42.5422	-5.4578
24	58	38	45.05	43.9662	-14.0339
25	38	35.74	49.9	48.1517	10.1517
26	46	43.8	41.4	40.8162	-5.1838
27	56	35.2	52.5	50.3955	-5.6045
28	34	41.4	43.6	42.7148	8.7148
29	50	28.77	46.3	45.0449	-4.9551
30	28	44	32.6	33.2218	5.2218
31	43	28.5	48	46.512	3.512
32	48	31.68	48	46.512	-1.488
33	40	39.38	53	50.827	10.827
34	47	33.59	50.05	48.2812	1.28115
35	47	28.72	45.7	44.5271	-2.4729
36	35	37.65	39.3	39.0039	4.0039

37	46	35.26	46.7	45.3901	-0.6099
38	45	32.42	53	50.827	5.827
39	43	26.82	35.6	35.8108	-7.1892
40	49	29.4	51.1	49.1873	0.1873
41	46	34.68	47.8	46.3394	0.3394
42	40	33.46	37	37.019	-2.981
43	42	37.72	33.2	33.7396	-8.2604
44	49	31.9	56.4	53.7612	4.7612
45	47	31.22	48.3	46.7709	-0.2291
46	57	34	56.3	53.6749	-3.3251
47	22	30.08	30.4	31.3232	9.3232
48	46	37.82	40.9	40.3847	-5.6153
49	21	50.19	31	31.841	10.841
50	40	39.38	43.9	42.9737	2.9737
51	28	28.57	34.2	34.6026	6.6026
52	35	37.65	41	40.471	5.471
53	36	37.73	33.2	33.7396	-2.2604
54	38	26.56	41.2	40.6436	2.6436
55	37	32.05	26.7	28.1301	-8.8699
56	43	34.45	37.3	37.2779	-5.7221
57	47	30.1	48.8	47.2024	0.2024
58	37	27.99	36.7	36.7601	-0.2399
59	36	38.54	37.3	37.2779	1.2779
60	21	25	29.8	30.8054	9.8054
61	41	27.81	41.8	41.1614	0.1614
62	37	33.27	40.8	40.2984	3.2984

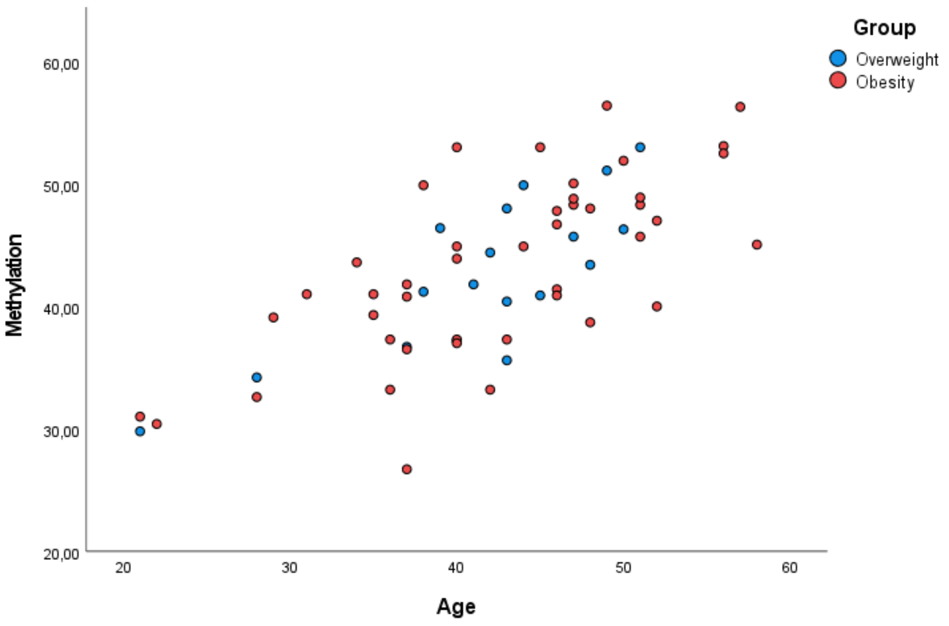

Figure S1. Correlation of DNAm levels of the *FHL2* CpG site cg06639320 with chronological age for women with overweight (blue) and obesity (red).
